# Glycemic Control and Management in Pharmacist-Led Diabetic Clinic vs. Physician-Led Diabetic Clinic

**DOI:** 10.3390/medicina58010014

**Published:** 2021-12-22

**Authors:** Sultan M. Alghadeer, Bashayr Alsuwayni, Abdulmohsen K. Almuwayjid, Mohammed S. Almadi, Abdullah M. Mubarak, Rawan M. bin Khunayn, Mohamed N. Al-Arifi

**Affiliations:** 1 Department of Clinical Pharmacy, College of Pharmacy, King Saud University, Riyadh 12372, Saudi Arabia; aalmuwayjid@gmail.com (A.K.A.); mohammed.salmadi@gmail.com (M.S.A.); malarifi@ksu.edu.sa (M.N.A.-A.); 2 Corporate of Pharmacy Services, King Saud University Medical City, Riyadh 12372, Saudi Arabia; balsuwayni@ksu.edu.sa; 3 Department of Basic Sciences, Prince Sultan College for EMS, King Saud University, Riyadh 12642, Saudi Arabia; abmubarak@ksu.edu.sa; 4 College of Medicine, Imam Mohammad Ibn Saud Islamic University (IMSIU), Riyadh 13317, Saudi Arabia; Rawan.mbk1@gmail.com

**Keywords:** diabetes, pharmacist-led clinic, physician-led clinic, hemoglobin A1c, glycemic control

## Abstract

*Background and Objectives*: Globally, diabetes Mellitus (DM) is a life-threatening disease that, if it remains uncontrolled, can lead to mortality or serious complications. Despite the noticeable benefits of clinical pharmacist in managing diabetes, some institutions in Saudi Arabia are reluctant to establish a pharmacist-led diabetic clinic for monitoring and follow-up. The objective of this study is to assess the glycemic control by comparing the reduction in hemoglobin A1c (HbA1c) percentage between patients followed in the pharmacist-led diabetic clinics vs. those followed in physician-led diabetic clinics. *Materials and Methods*: A retrospective observational study with a 12-month follow-up were used to detect the difference in the glycemic control by comparing the reduction in HbA1c percentage from the baseline, and average changes in HbA1c, fasting blood glucose (FBG), blood pressure (BP), and lipid panel between the two groups. The level of self-care was assessed by Summary of Diabetes Self-Care Activities (SDSCA) Questionnaire. *Results*: The study involved 52 patients who visited the diabetic clinic at a community teaching hospital. Exactly 24 patients were followed by the pharmacist-led diabetic clinics, while 28 were followed by physician-led diabetic clinics. HbA1c baseline was 8.7% and 8.4% for pharmacist and physician, respectively. The average difference in HbA1c for the pharmacist-led diabetic clinics vs. the physician-led diabetic clinics was not statistically significant (8.67 vs. 8.56; *p* = 0.77). Moreover, no difference in the glucose profile, lipid panel, and blood pressure were seen between the two groups. However, the median HbA1c change from baseline between the two groups significantly favored the pharmacist-led clinic (0.7 vs. 0.003; *p* = 0.04).The average of responses in all four aspects of the SDSCA (diet, exercise, blood sugar testing, and foot care) was also higher among patients in the pharmacist-led diabetic clinic. *Conclusions*: Pharmacist-led diabetic clinics for glycemic control and follow-up showed efficient results that encourage the comprehensive and integral inter-professional patient care.

## 1. Introduction

Statistics show that Saudi Arabia is at the second and seventh rank in the rate of diabetes in the Middle East and the world, respectively, with approximately 7 million of its people having diabetes, and more than 3 million having a significant risk for developing diabetes [1]. These facts make the diabetes disease a growing global health concern [2]. Therefore, various concerned organizations, such the American Diabetes Association (ADA), emphasize multidisciplinary work to apply their standards of care for diabetic patients [3]. Accordingly, the need for more frequent follow-ups and management is increasing, which might be overwhelming, considering the number of specialized physicians available in Saudi Arabia. However, the involvement of clinical pharmacists in diabetes management to collaboratively assist in caring for those patients remains underutilized [3,4,5]. As result, the multidisciplinary management of diabetes is becoming essential.

A multidisciplinary healthcare team is a concept that has been developed to promote health care delivery to patients. It is a team consisting of a group of professional health care providers from different specialties collaboratively making decisions towards improving the patient’s health care experience. A multidisciplinary diabetes health care delivery model, which includes clinical pharmacists running an ambulatory care clinic for managing, and/or following, diabetic patients who are evaluated nationally, and/or internationally, has frequently shown improvements in diabetes-related health outcomes, including with hemoglobin A1c (HbA1c), blood glucose, blood pressure, medication adherence, and health-related quality of life [2,3,4,6,7].

The clinical participation of pharmacist has shown an improvement in the health-related quality of life, medication adherence, number of hospital admissions, and severity of related diseases in several chronic conditions such chronic obstructive pulmonary disease (COPD) and benign prostatic hypertrophy (BPH). Importantly, the value of clinical pharmacists in diabetic care was remarkable by giving patient education, adherence support, medication monitoring, and aid with goal achievement, with other services. Multidisciplinary diabetes health care delivery models that have included clinical pharmacists running an ambulatory care clinic for managing/following diabetic patients have frequently shown improvements in HbA1c, blood glucose, blood pressure, medication adherence, and health-related quality of life [6,7,8,9]. Additionally, the pharmacist-led diabetic clinic can help in decreasing the burden of physicians-led diabetic clinics, optimizing the patient care, perfecting the administration of insulin, and, in turn, ensuring effective long-term management of high blood glucose [10].

Despite the noticeable benefits of clinical pharmacists in managing diabetes, some institutions in Saudi Arabia are reluctant to establish a pharmacist-led diabetic clinic for monitoring and follow-up. The absence of local data might contribute to such hesitation. HbA1c is a marker that is widely used in chronic glycemic patients, it reflects the average blood glucose levels over a 2- to 3-month period, and this test plays a major role in the management of the patient with diabetes. The aim of this study is to assess the difference in diabetic management utilizing HbA1c in clinics managed by clinical pharmacists vs. clinics managed by physicians.

## 2. Materials and Methods

A retrospective chart review study with 12-month follow-up from 4/2018 to 12/2020 was used to investigate the difference in management of diabetes mellitus between the patients followed in the pharmacist-led diabetic clinics vs. those followed in physician-led diabetic clinics. The study was conducted at the diabetes management clinics at King Saud University Medical City (KSUMC). KSUMC is a governmental public medical city that is providing patient care regardless of their insurance coverage. The diabetes management clinics are established for follow-up management and monitoring after the initial diagnosis of diabetes. These clinics are either covered by primary care physicians or clinical pharmacists who have sufficient training and/or a certain certificate for diabetes management. Prior to each visit to these clinics, a pre-visit planning checklist to determine the scope of services is conducted. At each visit, the clinician either (physician or pharmacist) collects subjective and objective data to assess: (1) the level of patient’s disease/condition; (2) the adherence with pharmacological, non-pharmacological, and self-management aspects of the therapy; (3) presence of complications or hospital admission due to condition or medication therapy; and (4) need for ancillary monitoring, prevention services, and nutritional or weight loss support. Based on the findings, the clinician will: (1) order indicated/appropriate laboratory tests; (2) recommend/modify existing pharmacological regimen for management of patient’s disease (s)/condition (s); (3) provide needed interventions and educate patients on self-management of their condition including the effective use of medication therapy; (4) refer the patient to other appropriate providers when needed; or/and (5) schedule a follow-up appointment. Our study was ethically approved by the health section in the institute of research board at KSUMC (IRB Project No. E-20-5144). Since the study is retrospective observational study, the consent was not required. The Arabic validated version of Summary of Diabetes Self-Care Activities (SDSCA) Questionnaire was used [11]. The permission to use was taken from the original developers through Oregon Research Institute [12].

The primary outcome was to assess the glycemic control by comparing the reduction in HbA1c percentage between two groups. The secondary outcomes include the comparison between the two groups in the biomedical parameters such the glucose profile, lipid panel, and blood pressure. Additionally, the level of self-care between the two comparable groups was evaluated using SDSCA Questionnaire. The SDSCA measure is a brief self-report questionnaire of diabetes self-management that includes 15 items assessing the following five aspects (domains) of the diabetes regimen: general diet (5 items), exercise (2 items), blood-glucose testing (2 items), foot care (5 items), and medication (1 item). For each item, the participant is asked about his/her diabetes self-care activities during the past 7 days. The mean of numbers of days for each item is calculated, and then the mean score for each domain is expressed.

All adult subjects diagnosed with diabetes who were referred to the diabetic follow-up clinics and seen exclusively by either physician-led or pharmacist-led clinic during the period of study were included in the study. Any patient who had been followed by both physician-led and pharmacist-led clinic during the period of study was excluded from the study.

Normally distributed continuous variables were presented as mean and percentage by using the Student’s t-test, while median and interquartile range (IQR) were presented for the non-normally distributed variables by using the Mann–Whitney U test. The Shapiro–Wilk test was used to assess for the normal distribution with value above 0.5 is considered normally distributed. For categorical variables, chi square test was used; however, if the assumption was violated, the Fisher exact test was utilized. Data were analyzed using SPSS (SPSS Inc., Chicago, IL, USA) software.

## 3. Results

Only 52 patients from a total of 294 patients who visited the diabetic clinic at King Khaled University Hospital (one of the three hospitals in KSUMC) between 9/2018 and 12/2020 met our inclusion criteria, and are included in the study. Of the total of 52, exactly 24 patients were followed by the pharmacist-led diabetic clinics, while 28 were followed by physician-led diabetic clinics. The baseline and social characteristics were very comparable between the two groups (Table 1 and Table 2). Median age was 63 for the both groups. Most patients were female (70.8% in the pharmacist-led clinic and 50% in physician-led clinic); Type 2 diabetes was the predominant type (95.8% and 92.9%, respectively). The median baseline HbA1c was almost the same for the comparable groups (8.7 vs. 8.4; *p =* 0.47). Other baseline characteristics are presented in Table 1. Most of our participants are married, and have university degree or higher with income ≥8000 Saudi Riyals. Details of the social characteristics are shown in Table 2.

**Figure 1:**
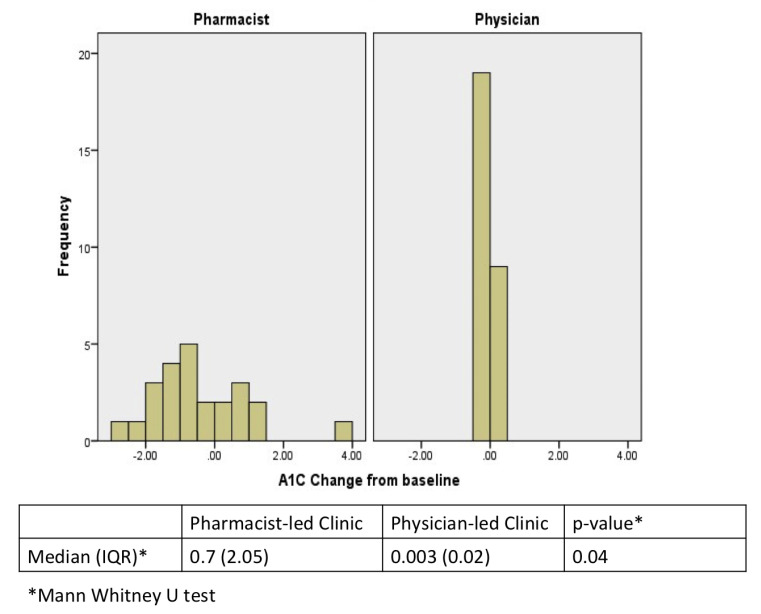
Median HbA1c change from baseline.

During a period of 12 months follow-up, the number of patients’ visits was significantly higher in the pharmacist-led clinic compared to physician-led clinic (*p =* 0.0005). However, the average difference in HbA1c for the pharmacist-led diabetic clinics vs. the physician-led diabetic clinics was not statistically significant (8.67 vs. 8.56; *p =* 0.77). Moreover, no difference in the glucose profile, lipid panel, and blood pressure were seen between the two groups (Table 3). However, the median HbA1c change from baseline between the two groups was significant, favoring the pharmacist-led clinic (0.7 vs. 0.003; *p =* 0.04) (Figure 1). Additionally, the pharmacist-led clinic changed the doses of DM medication for their patients more frequent compared to the physician-led clinic (*p =* 0.16) (Table 4).

**Figure 2:**
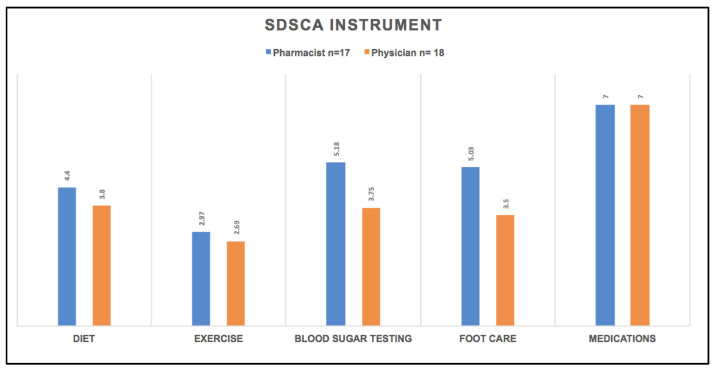
Summary of Diabetes Self-Care Activities (SDSCA).

The patients were contacted to complete summary of diabetes self-care activities questionnaire (SDSCA). Thirteen, and four, out of the fifty-two enrolled patients did not answer and refused to participate, respectively; seven participants (one refused and six did not answer) were followed in the pharmacist-led diabetic clinics, while ten participants (three refused and seven did not answer) were in the physician-led diabetic clinics. The average of responses in all four aspects of the questionnaire (diet, exercise, blood sugar testing, and foot care) was higher among patients in the pharmacist-led diabetic clinic compared with those in the physician-led diabetic clinics (Figure 2). Compliance with medication usage was the same between both groups. Smoking was reported in one patient at pharmacist-led diabetic clinic with daily reported average of 45 cigarettes per day, while two patients were smokers in physician-led diabetic clinics group with daily reported average of 20 and 15 cigarettes per day.

## 4. Discussion

Although our study shows no difference in the average HbA1c between pharmacist-led clinic vs. physician-led clinic by the end of follow-up (8.67 vs. 8.57, *p =* 0.77), the HbA1c change from baseline was significantly higher among patients who followed at the pharmacist-led clinic (0.7 vs. 0.003; *p =* 0.04). Such findings demonstrate the capability of pharmacists to manage the diabetes; and thus, similar studies with relatively similar findings were conducted. A study conducted in the United States compared the diabetes management for 82 patients between pharmacist- and physician-led clinics in terms of HbA1c reduction, hospitalization, and emergency admission [13]. They found that patients followed in the pharmacist-led clinic had significant HbA1c (1.63% vs. 1.53%, *p* < 0.0001) and emergency admission reduction (27 vs. 8 patients, *p =* 0.049), and non-significant, but decline in hospitalization (6 vs. 10 patients). Similarly, pharmacist interventions for diabetic patients were assessed against the usual care in community outpatient clinics (n = 782), and found that patients subject to clinical pharmacist services had significant HbA1c reduction (*p* < 0.5) and non-significant, but lower emergency visits and related hospitalization [14]. Additionally, a pharmacist-managed program was established in a primary clinic in the United States, and this program was assessed against the usual care. Around 980 patients enrolled in the pharmacist-managed program were compared to the actual number of patients exposed to the unusual care in the primary clinic to assess glycemic control [15]. The goal of HbA1c < 8% was achieved by the pharmacist-managed program at 3 months and 6 months with odd ratios of 2.44 (*p* < 0.0001) and 1.32 (*p* = 0.007), respectively. Moreover, the program showed a significant change in the baseline HbA1c compared with the usual care at 3 months (−0.95% vs. −0.54%, *p* < 0.0001), and 6 months (−1.19% vs. −0.99%, *p =* 0.008). Furthermore, a prospective study was conducted in Saudi Arabia at a community teaching hospital to evaluate the impact of pharmacists on patient-related health outcomes in diabetes management clinic in terms of HbA1c level. It showed a significant improvement by 1.2% (*p* = 0.0004) in terms of HbA1c reduction [4].

The number of follow-up visits that are usually associated with various interventions such as patient education, medication management, and dosage adjustment were significantly higher in the pharmacist-led clinic (median 5 vs. 3 visits, *p* < 0.0001). Moreover, the number of dosage adjustments was higher in the pharmacist-led clinic (*p =* 0.016). These results are consistent with other studies that showed an increase in pharmacists’ interventions compared to the usual case for diabetic patients. Benedict AW et al. reported that there were almost three pharmacist interventions per patient, mostly either the phone call (65%) or in-clinic (34%) [14]. Additionally, Schultz JL et al. found that patients in the pharmacist-led clinic had higher “dosage adjustment of injectable medication” (41%) vs. those in the physician-led clinic (21%), while “dosage adjustment of oral medication” were similar for both groups (6%) [15].

Although the summary of diabetes self-care activities (SDSCA) questionnaire did not show a significant difference between the two groups, the pharmacist-led clinic had slightly better outcome management in terms of diet and exercise, blood sugar testing, foot care assistances, and encouragements, which are highly recommended in order to improve diabetes and prevent or minimize the chances of developing diabetes complications by empowering patient self-management. Our study showed a positive impact of the collaborative practice agreement in only a 12-month evaluation period. Both groups showed improvements in overall diabetes-related outcomes. This study showed a successful and comparable level of care that is not limited to achieving the patient’s targeted HbA1c, but extended to assessing other diabetes-related risks, and the performing of the guidelines’ recommended preventive measures, whenever possible. This will open up more confident future collaborations towards better patient care, and support the multidisciplinary concept for diabetes management.

Our study has some limitations. It is a retrospective observational study that included small number of patients, mostly type 2 DM, from one healthcare center. The strict inclusion criteria for having only patients who followed-up by either physicians or pharmacist, and the retrospective nature of study that hindered the allocation of subjects into each group might contribute to the small numbers of patients. This also might be attributed to having the clinical pharmacist’s service being provided for only half a day per week, since it is considered relatively new. Regardless of small number of patients, the study results are consistent with results from other studies in terms of HbA1c improvement. Other than dosages adjustments, our study was unable to specify the interventions from either group. However, each group is assumed to have provided the same level of education regarding diet, exercise, foot care, home glucose monitoring, and medication adherence, but there is not enough documentation regarding these interventions. The free of charge services for all patients in the diabetes management clinic could contribute to insufficient documentation.

## 5. Conclusions

The integration of clinical pharmacists in diabetes and/or other chronic disease management within a collaborative practice agreement will enhance medication utilization, improve disease-related outcomes, and correspondingly reduce cost and promote the overall patient experience.

## Figures and Tables

**Table 1:** Baseline characteristics.

Characteristic	Pharmacist-Led Diabetic Clinics n = 24	Physician-Led Diabetic Clinics n = 28	*p*-Value *
Sex			0.16
Male	7 (29.2%)	14 (50%)	
Female	17 (70.8%)	14 (50%)	
Age (Years), (median, IQR)	63.5 (8.5)	63 (19.5)	0.81
DM Type			1
1	1 (4.2%)	2 (7.1%)	
2	23 (95.8%)	26 (92.9%)	
Average BMI Classification			1
Below 18.5 underweight	0	0	
18.5–24.9 normal	3 (12.5%)	4 (14.3%)	
25–29.9 Over weight	8 (33.3%)	9 (32.1%)	
Over 30 Obesity	13 (54.2%)	15 (53.6%)	
Baseline Lipid Panel			
LDL (mean, SD)	2.2 ± 0.76	2.11 ± 0.81	0.68
Cholesterol (median, IQR)	4.27 (0.88)	3.83 (1.33)	0.51
HDL (median, IQR)	1.15 (0.5)	1.2 (0.28)	0.88
Triglyceride (mean, SD)	1.56 ± 0.96	1.44 ± 0.59	0.5
Baseline HbA1c (median, IQR)	8.7 (2.28)	8.4 (1.58)	0.47
Baseline FBG (Mean, SD)	10.37 ± 4.35	8.93 ± 4.23	0.18
Comorbidity +			
Dyslipidemia	16	4	
HTN	18	19	
Hypo-Hyperthyroidism	5	2	
Cardiac Patient	3	2	
Other ^	17	13	
Baseline BP			
SBP (Mean, SD)	132.25 ± 16.62	134.17 ± 20.28	0.63
DBP (Mean, SD)	71.66 ± 14.75	75.43 ± 16.75	0.3

(*) Significant result at ɑ = 0.05; (+) The comorbidities were calculated based on the number of appearances in patient groups; (^) This include several diseases should be explained in the result section. Abbreviations: BMI, body mass index; BP, blood pressure; DBP, diastolic blood pressure; DM, diabetes; FBG, fasting blood glucose; HbA1c, hemoglobin A1c; HDL, high-density lipoprotein; LDL, low-density lipoprotein; SBP, systolic blood pressure.

**Table 2:** Social demographics.

Social Demographics	Pharmacist-Led Diabetic Clinics n = 17	Physician-Led Diabetic Clinics n = 18	*p*-Value
Social Status n = 35 (17 Patients Refused to Participate)			0.69
Single	4	3	
Married	13	15	
Education Level n = 35 (17 Patients Refused to Participate)			0.59
Uneducated	3	0	
Primary School	1	1	
Intermediate School	2	3	
Secondary School	2	1	
University	5	5	
Postgraduate education	4	8	
Family Monthly Income n = 35 (17 Patients Refused to Participate)			0.54
Less than 4000 SR	2	0	
From 4000 to 8000 SR	2	3	
From 8000 to 15,000 SR	6	4	
From 15,000 to 23,000 SR	2	6	
More than 23,000 SR	5	5	
Number of Family Members n = 35 (17 Patients Refused to participate)			0.44
0–2	1	2	
3–4	1	3	
5–6	8	4	
More than 6	7	9	

Abbreviations: SR, Saudi Riyal.

**Table 3:** Difference in HbA1c and other biomedical parameters.

Characteristics	Pharmacist-Led Diabetic Clinics n = 24	Physician-Led Diabetic Clinics n = 28	*p*-Value
Number of Visits (Median, IQR)	5 (1.8)	3 (2)	0.0005
Number of Visits			0.0005
2–3	3 (12.5%)	18 (64.3%)	
4–6	17 (70.8%)	10 (35.7%)	
More than 6	4 (16.7%)	0	
Average Lipid Panel			
LDL (median, IQR)	2.41 (1.4)	2.07 (0.63)	0.24
Cholesterol (median, IQR)	4.43 (1.47)	3.97 (0.84)	0.11
HDL (mean, SD)	1.28 ± 0.51	1.19 ± 0.4	0.39
Triglyceride (median, IQR)	1.65 (1.19)	1.38 (0.84)	0.42
Average HbA1c (median, IQR)	8.67 (1.45)	8.56 (1.82)	0.77
Average FBG (median, IQR)	9.51 (3.57)	9.14 (3.18)	0.53
Average BP			
SBP (Mean, SD)	134.63 ± 10.06	136.38 ± 15.51	0.64
DBP (Mean, SD)	71.64 ± 7.8	75.45 ± 9.65	0.13

Abbreviations: BP, blood pressure; DBP, diastolic blood pressure; FBG, fasting blood glucose; HbA1c, hemoglobin A1c; HDL, high-density lipoprotein; LDL, low-density lipoprotein; SBP, systolic blood pressure.

**Table 4:** Changes in diabetic medication doses.

Number of Changed Doses per Patient	Pharmacist-Led Clinics	Physician-Led Clinics	Total
0	3	7	10
1	6	10	16
2	4	10	14
3	1	1	2
4	4	0	4
5	4	0	4
6	1	0	1
7	1	0	1
Total	24	28	52

Notes: *p* = 0.016 (Fisher exact test).
